# Integrating AI-generated content tools in higher education: a comparative analysis of interdisciplinary learning outcomes

**DOI:** 10.1038/s41598-025-10941-y

**Published:** 2025-07-16

**Authors:** Zhang Yan, Tang Qianjun

**Affiliations:** 1 Law and Public Management School, Leshan Normal University, Leshan, 614000 Sichuang China; 2 Educational Science School, Leshan Normal University, Leshan, 614000 Sichuang China

**Keywords:** AIGC tools, Higher education, Interdisciplinary learning, Digital pedagogy, Educational policy, AI–Human collaboration, Computer science, Information technology, Scientific data

## Abstract

This study examines the integration of artificial intelligence-generated content (AIGC) tools across disciplines in higher education settings. Using a mixed-methods approach, we analyzed implementation patterns and learning outcomes across humanities, STEM, and social sciences programs at multiple institutions. Findings revealed a 37% increase in interdisciplinary project outcomes (measured by collaborative problem-solving scores, cross-domain knowledge integration ratings, and peer evaluation metrics) when AIGC tools were strategically implemented, with significant variations in effectiveness based on implementation approach. While these technologies demonstrated substantial value in breaking down disciplinary silos and accommodating diverse learning preferences, challenges emerged regarding algorithmic bias, digital equity, and maintenance of discipline-specific skills. This research contributes to educational theory by proposing a revised framework for AI-human collaboration in knowledge production. We conclude with policy recommendations for governance frameworks that balance innovation with academic integrity, emphasizing faculty co-design approaches and the establishment of cross-disciplinary communities of practice.

## Introduction

### Background and current status

The rapid development of artificial intelligence technologies has profoundly impacted various sectors, with education emerging as a significant arena for artificial intelligence generated content (AIGC) implementation^1^. Higher education institutions worldwide are increasingly integrating AIGC tools such as ChatGPT, DALL-E, and Midjourney into teaching methodologies, research practices, and administrative functions^2^. These technologies, powered by large language models (LLMs) and diffusion-based image generators, have demonstrated remarkable capabilities in content creation, problem-solving, and simulation that were previously unattainable through conventional educational technologies^3^.

The adoption rate of AIGC tools in higher education can be quantified using the Technology Acceptance Model (TAM):

$$\:A=\frac{PU\times\:PEOU}{R}$$

where $\:A$ represents adoption rate, $\:PU$ denotes perceived usefulness, $\:PEOU$ indicates perceived ease of use, and $\:R$ signifies institutional resistance^4^.

### Research significance and objectives

Despite growing implementation, systematic evaluation of AIGC tools’ impact on interdisciplinary learning outcomes remains underdeveloped^5^. This research gap is particularly concerning as institutions invest significant resources in AI integration without comprehensive understanding of differential outcomes across disciplines. The educational efficacy of these tools varies substantially across disciplines due to varying knowledge structures, pedagogical approaches, and evaluation metrics^6^.

The relationship between AIGC implementation and learning outcomes can be expressed as:

$$\:LO=\alpha\:\times\:AIGC+\beta\:\times\:DP+\gamma\:\times\:\left(AIGC\times\:DP\right)+ϵ$$

where $\:LO$ represents learning outcomes, $\:AIGC$ denotes the level of AI-generated content tool implementation, $\:DP$ represents discipline-specific pedagogical approaches, and $\:ϵ$ signifies unaccounted variables^7^.

This study aims to conduct a comparative analysis of AIGC tool integration across five distinct academic disciplines: engineering, humanities, business, life sciences, and fine arts. By measuring pedagogical effectiveness through a multi-dimensional assessment model, we can derive the optimization function:

$$\:E=\sum\:_{i=1}^{n}{w}_{i}\times\:{O}_{i}\times\:{D}_{i}$$

where $\:E$ represents educational effectiveness, $\:{w}_{i}$ denotes the weight of outcome measure $\:i$, $\:{O}_{i}$ represents the measured outcome, and $\:{D}_{i}$ indicates discipline-specific contextual factors^8^.

### Structure and scope

This paper is organized as follows: Section II reviews relevant literature on AIGC applications in educational contexts; Section III details the methodological framework employed; Section IV presents the comparative analysis findings across disciplines; Section V discusses implications for educational policy and practice; and Section VI concludes with recommendations for future research directions and implementation strategies.

The significance of this research extends beyond academic interest, addressing pressing challenges faced by higher education institutions regarding AI integration. As AIGC tools become increasingly sophisticated and accessible, developing evidence-based approaches to their implementation becomes essential for maximizing educational benefits while mitigating potential drawbacks associated with automated content generation, academic integrity concerns, and differential technological access.

### Types and characteristics of AI-Generated content tools

#### Text generation tools

Text generation tools, primarily based on large language models (LLMs), represent the most widely adopted AIGC technologies in higher education contexts^1^. These systems utilize transformer architectures with self-attention mechanisms to model sequential data, as represented by the equation:

$$\:Attention\left(Q,K,V\right)=softmax\left(\frac{Q{K}^{T}}{\sqrt{{d}_{k}}}\right)V$$

where Q, K, and V represent query, key, and value matrices respectively, and $\:{d}_{k}$ denotes the dimension of the key vectors^2^. Current implementations such as GPT-4 and Claude demonstrate sophisticated capabilities in content generation, summarization, and text transformation that support diverse pedagogical applications^3^. Wang et al. conducted a comprehensive analysis of 78 higher education institutions, finding that text-based AIGC tools were integrated into 64% of humanities courses but only 37% of STEM-focused curricula^4^.

#### Image generation tools

Diffusion models have emerged as the dominant paradigm for image generation, operating through the iterative denoising process described by:

$$\:{x}_{t-1}=\frac{1}{\sqrt{{\alpha\:}_{t}}}\left({x}_{t}-\frac{1-{\alpha\:}_{t}}{\sqrt{1-{\overset{\lower0.5em\hbox{$\smash{\scriptscriptstyle\leftharpoonup}$}} {\alpha }{\alpha\:}}_{t}}}{\epsilon}_{\theta\:}\left({x}_{t},t\right)\right)+{\sigma\:}_{t}z$$

where $$\:{x}_{t}$$ represents the image at noise level $$\:t$$, $$\:{\alpha\:}_{t}$$ and $$\:{\overset{\lower0.5em\hbox{$\smash{\scriptscriptstyle\leftharpoonup}$}} {\alpha }{\alpha\:}}_{t}$$ are noise scheduling parameters, and $$\:{\epsilon}_{\theta\:}$$ is the learned noise prediction function^5^. Platforms such as DALL-E 3, Midjourney, and Stable Diffusion have demonstrated significant utility in design courses, visual arts education, and architectural visualization^6^. The integration of image generation tools presents unique challenges regarding attribution and originality assessment, with Martinez identifying significant disparities in institutional policies governing their use^7^.

#### Audio generation tools

Contemporary audio generation systems employ neural vocoding techniques that transform spectrograms into waveforms through the following process:

$$\:p\left(x|c\right)=\prod\:_{t=1}^{T}p\left({x}_{t}|{x}_{1:t-1},c\right)$$

where $\:x$ represents the audio waveform, $\:c$ denotes the conditioning information, and $\:p\left({x}_{t}|{x}_{1:t-1},c\right)$ is modeled using autoregressive neural networks^8^. Tools such as Bark, AudioLM, and MusicLM enable the creation of speech, music, and environmental sounds with applications in language learning, music composition, and audio production courses^9^. Research by Chen et al. indicates that audio AIGC tools improved student engagement by a factor of 1.8 compared to traditional instructional methods in language acquisition courses^10^.

#### Educational implications and limitations

The educational effectiveness of AIGC tools can be quantified using a composite index that incorporates learning outcomes, engagement metrics, and skill development:

$$\:{E}_{AIGC}={\omega\:}_{1}L+{\omega\:}_{2}G+{\omega\:}_{3}S-{\omega\:}_{4}B$$

where $\:L$ represents learning outcome improvement, $\:G$ denotes engagement gains, $\:S$ indicates skill development, $\:B$ represents bias or accuracy concerns, and $\:{\omega\:}_{1-4}$ are contextual weighting factors^11^. Despite their potential, AIGC tools face significant limitations in higher education contexts, including accuracy concerns, potential reinforcement of biases, and challenges in developing critical evaluation skills among students^12^. The effective implementation of these technologies requires thoughtful pedagogical frameworks that emphasize critical evaluation, transparent attribution, and complementary application alongside traditional learning methodologies.

### Technology integration models in higher education

The successful integration of emerging technologies, including AIGC tools, into higher education requires structured theoretical frameworks to guide implementation. Several established models offer valuable insights into this process, each with distinct approaches to technology adoption and pedagogical integration.

#### Technology integration models and their application to AIGC

The Technological Pedagogical Content Knowledge (TPACK) framework emphasizes the complex interplay between three knowledge domains: content, pedagogy, and technology^11^. The effectiveness of technology integration can be expressed through the following relationship:

$$\:{E}_{TPACK}=\alpha\:\left(CK\right)+\beta\:\left(PK\right)+\gamma\:\left(TK\right)+\delta\:\left(CK\times\:PK\times\:TK\right)$$

Where α, β, γ, and δ represent weighting factors for Content Knowledge (CK), Pedagogical Knowledge (PK), and Technological Knowledge (TK) respectively^12^.

The Substitution, Augmentation, Modification, Redefinition (SAMR) model provides a progression framework for technology adoption, moving from enhancement to transformation^13^. In the context of AIGC integration, this transition can be represented as:

$$\:{T}_{impact}=\sum\:_{i=1}^{4}{S}_{i}\times\:{W}_{i}$$

Where T represents technological impact, S represents each SAMR stage, and W represents the corresponding weight of pedagogical transformation^14^.

The Technology Acceptance Model (TAM) focuses on perceived usefulness (PU) and perceived ease of use (PEOU) as determinants of adoption^15^. The behavioral intention to use AIGC tools can be modeled as:

$$\:BI={\beta\:}_{1}\left(PU\right)+{\beta\:}_{2}\left(PEOU\right)+\epsilon$$

Where BI represents behavioral intention, and β1 and β2 are regression coefficients^16^.

The ADDIE model (Analysis, Design, Development, Implementation, Evaluation) provides a systematic approach to instructional design that can be adapted for AIGC integration^17^. The model’s effectiveness can be quantified through:

$$\:{E}_{ADDIE}=\frac{\sum\:_{i=1}^{5}{P}_{i}\times\:{w}_{i}}{5}$$

Where P represents performance in each ADDIE phase and w represents phase weight^18^.

Recent research indicates that these models must evolve to address the unique characteristics of AIGC tools, including their generative capabilities, ethical considerations, and potential for personalized learning experiences^19^. As shown in Table 1, these educational technology integration models differ in their core concepts and implications for AIGC integration. Digital transformation in higher education has accelerated the need for frameworks that specifically address AI-enhanced learning environments^20^.

**Table 1:** Comparison of educational technology integration Models.

Model name	Core concepts	Application characteristics	Implications for AIGC integration
TPACK	Intersection of content, pedagogical, and technological knowledge	Holistic approach emphasizing knowledge domains interaction	Requires faculty development in AI literacy and pedagogical applications
SAMR	Progressive stages from substitution to redefinition	Task-oriented transformation framework	Provides pathway for evolving AIGC implementation from simple to transformative uses
TAM	Perceived usefulness and ease of use as adoption predictors	Focus on user acceptance factors	Highlights importance of user-friendly interfaces and demonstrated value in AIGC tools
ADDIE	Systematic instructional design process	Structured development approach	Offers systematic framework for AIGC-enhanced course design
Community of inquiry	Social, teaching, and cognitive presence	Emphasizes collaborative knowledge construction	Guides integration of AIGC tools to support meaningful educational interactions

The evolution of these models reflects the changing landscape of educational technology integration. While early models focused primarily on hardware and software adoption, contemporary frameworks increasingly address the socio-technical systems and pedagogical transformations enabled by advanced technologies. The rapid development of AIGC tools presents both challenges and opportunities for these theoretical frameworks, necessitating adaptations that account for AI’s unique capabilities and limitations.

### Cross-disciplinary learning and AIGC technology correlation research

Cross-disciplinary learning has emerged as a critical educational paradigm for developing complex problem-solving capabilities in the 21st century knowledge economy^1^. The theoretical foundation for cross-disciplinary learning can be traced to constructivist learning theory, which emphasizes knowledge construction through authentic problem-solving experiences across multiple domains^2^. Recent educational frameworks have formalized this approach through the Integrated Cross-disciplinary Learning Index (ICLI), which can be expressed as:

$$\:ICLI=\sum\:_{i=1}^{n}{w}_{i}\left({D}_{i}\times\:{C}_{i}\right)$$

Where $\:{D}_{i}$ represents domain knowledge integration, $\:{C}_{i}$ indicates cognitive transfer across disciplines, and $\:{w}_{i}$ denotes the weighted importance of each disciplinary component^3^.

The integration of AIGC technologies into cross-disciplinary learning environments introduces new dynamics in knowledge co-construction and representation capabilities^4^. Studies indicate that AIGC tools can enhance cross-boundary thinking by providing computational support for domain knowledge synthesis through the Cross-domain Knowledge Synthesis Factor (CKSF):

$$\:CKSF=\frac{KI\times\:CT}{BD}$$

Where KI represents knowledge integration, CT denotes creative transformation, and BD indicates boundary-crossing difficulty^5^.

Contemporary research on AIGC applications in cross-disciplinary education demonstrates promising results across various institutional contexts^6^. As shown in Table 2, various cross-disciplinary learning models can be supported by different AIGC strategies to enhance educational outcomes. A meta-analysis of 27 studies revealed that AIGC-enhanced cross-disciplinary projects improved student learning outcomes by an average effect size of 0.68, particularly in complex problem-solving scenarios^7^. This improvement can be quantified through the Interdisciplinary Learning Enhancement Rate (ILER):

$$\:ILER=\frac{{P}_{post}-{P}_{pre}}{{P}_{max}-{P}_{pre}}\times\:100\text{\%}$$

Where $\:{P}_{post}$ and $\:{P}_{pre}$ represent post-intervention and pre-intervention performance, respectively, and $\:{P}_{max}$ indicates maximum possible performance^8^.

**Table 2:** Cross-disciplinary learning models and AIGC support Strategies.

Cross-disciplinary model type	Learning characteristics	AIGC support strategies
Multidisciplinary integration	Preserves disciplinary boundaries while addressing common themes	Content generation across multiple domains; Automated synthesis of multidisciplinary resources
Interdisciplinary problem-based learning	Integrates methodologies from multiple disciplines to solve authentic problems	Complex scenario generation; Adaptive content scaffolding; Alternative perspective simulation
Transdisciplinary knowledge creation	Transcends disciplinary boundaries to create new conceptual frameworks	Knowledge gap identification; Cross-domain concept mapping; Emergent pattern recognition
Convergence learning	Synthesizes knowledge and methods from diverse fields into unified frameworks	Real-time knowledge integration; Cross-disciplinary translation; Systems-level modeling

Despite promising developments, significant research gaps persist in understanding the optimal integration mechanisms for AIGC tools in cross-disciplinary learning^9^. Current studies predominantly focus on short-term interventions rather than longitudinal impacts on knowledge transfer and disciplinary identity development^10^. Additionally, research methodologies require greater standardization to facilitate meaningful cross-study comparisons and generalizable design principles for AIGC-enhanced cross-disciplinary learning environments.

## Research methods

### Research design and framework

This study employs a mixed-methods research design to investigate the integration of AI content generation tools across disciplinary contexts in higher education. The quantitative strand employed pre-post experimental design with validated instruments measuring learning outcomes (*n* = 1099), technology acceptance surveys (32-item Likert scale), and learning analytics from LMS platforms. The qualitative strand utilized semi-structured interviews (*n* = 42), focus groups (*n* = 8), and classroom observations (86 sessions) using standardized protocols. The research framework is grounded in the educational technology acceptance model proposed by Johnson et al., which emphasizes the interplay between technological affordances and pedagogical objectives^1^. The design incorporates both quantitative and qualitative approaches to address the multifaceted nature of AI tool integration and learning outcomes measurement.

The primary research questions guiding this investigation are: (1) How do AI content generation tools affect student learning outcomes across different disciplines? (2) What factors moderate the effectiveness of AI tool integration in educational contexts? and (3) What cross-disciplinary patterns emerge in student engagement with AI-generated content? These questions informed the development of three hypotheses:

#### H1

Students in disciplines with structured knowledge domains will demonstrate higher learning gains from AI tool integration than those in less structured domains.

#### H2

The relationship between AI tool usage and learning outcomes is moderated by instructor technological pedagogical content knowledge (TPACK)

#### H3

Cross-disciplinary variation in learning outcomes follows the function: $LO=\alpha\:+{\beta\:}_{1}\left(A{I}_{usage}\right)+{\beta\:}_{2}\left(Dis{c}_{structure}\right)+{\beta\:}_{3}\left(A{I}_{usage}\times\:Dis{c}_{structure}\right)+\epsilon\:$

The mixed-methods approach was selected because it allows for triangulation of findings through complementary data sources, enhancing validity and providing richer insights than either method alone^2^. The quantitative strand examines causal relationships between AI tool usage and measured learning outcomes, while the qualitative strand explores the contextual factors and lived experiences that shape these relationships. The research design matrix, presented in Table 3, outlines how different research questions are addressed through various data types, collection methods, analysis techniques, and quality control measures.

Variables were operationalized following Martínez-Argüelles’ framework for educational technology assessment^3^. The independent variable (AI tool integration) was measured through usage frequency, diversity of applications, and depth of implementation. Dependent variables (learning outcomes) were assessed through standardized instruments validated by Chen and colleagues^4^. Moderating variables included discipline structure, instructor TPACK, institutional support, and student digital literacy.

The comparative analysis framework draws on Thompson’s cross-disciplinary educational technology taxonomy, which provides standardized dimensions for comparing technology implementation across knowledge domains^5^. This taxonomy was adapted to include AI-specific dimensions such as content autonomy, feedback mechanisms, and creative contribution.

**Table 3:** Research design matrix.

Research questions	Data types	Collection methods	Analysis techniques	Quality control measures
How do AI tools affect learning outcomes across disciplines?	Quantitative: Test scores, course grades, engagement metrics; Qualitative: Reflective journals	Pre-post assessments, LMS analytics, semi-structured interviews	Hierarchical linear modeling, ANCOVA, thematic analysis	Instrument validation, inter-rater reliability, member checking
What factors moderate AI effectiveness?	Quantitative: Survey responses; Qualitative: Observational data	Validated scales, classroom observations, instructor interviews	Structural equation modeling, moderation analysis, constant comparative method	Pilot testing, triangulation, peer debriefing
What cross-disciplinary patterns emerge?	Quantitative: Comparative metrics; Qualitative: Case studies	Cross-sectional surveys, longitudinal tracking, focus groups	Cluster analysis, discriminant analysis, cross-case synthesis	Mixed methods validation, audit trail, reflexivity
What implementation practices maximize benefits?	Quantitative: Implementation metrics; Qualitative: Best practice examples	Implementation checklists, expert interviews, document analysis	Regression analysis, pattern matching, content analysis	Expert review, methodological triangulation, thick description

The research proceeded in three sequential phases: (1) baseline assessment and implementation, (2) intervention and data collection, and (3) analysis and cross-disciplinary comparison. This phased approach facilitated iterative refinement of the research instruments and ensured systematic comparison across disciplines while maintaining contextual awareness of discipline-specific factors.

### Research objects and sampling methods

The research participants were selected from 15 higher education institutions across four geographical regions, representing a diverse spectrum of institutional types including research universities, teaching-focused universities, and specialized colleges. A multi-stage stratified random sampling technique was employed to ensure the representativeness of the sample^17^. The sample size for each discipline stratum was determined using the following formula:

$$\:{n}_{h}=\frac{{N}_{h}}{N}\times\:n$$

Where $\:{n}_{h}$ represents the sample size for stratum $\:h$, $\:{N}_{h}$ is the population size for stratum $\:h$, $\:N$ is the total population size, and $\:n$ is the total sample size^18^.

The confidence level for sampling was established at 95%, with a margin of error of ± 3.5%. The minimum required sample size was calculated using:

$$\:n=\frac{{z}^{2}\times\:p\times\:\left(1-p\right)}{{e}^{2}}$$

Where $\:z$ is the z-score (1.96 for 95% confidence level), $\:p$ is the estimated proportion (0.5 was used to maximize sample size), and $\:e$ is the margin of error^19^.

To address potential non-response rates, the final sample size was adjusted using:

$$\:{n}_{adjusted}=\frac{n}{\left(1-r\right)}$$

Where $\:r$ represents the anticipated non-response rate, estimated at 20% based on previous similar studies^20^.

Quota sampling was applied within each institution to ensure proportional representation across academic disciplines, faculty ranks, and student academic levels. Table 4 presents the final research sample composition across different discipline categories, including the number of schools, faculty, students, gender ratio, and technology proficiency.

**Table 4:** Research sample Composition.

Discipline category	Schools (*n*)	Faculty (*n*)	Students (*n*)	Gender ratio (M: F)	Technology proficiency*
STEM	6	87	342	56:44	4.2 ± 0.6
Humanities	4	65	298	42:58	3.7 ± 0.8
Social Sciences	3	58	276	47:53	3.9 ± 0.7
Health Sciences	2	42	183	39:61	3.8 ± 0.5
Total	15	252	1099	47:53	3.9 ± 0.7

*Technology proficiency measured on a 5-point Likert scale (1 = Very Low, 5 = Very High).

Faculty participants (*n* = 252) represented diverse academic ranks, with 28% full professors, 36% associate professors, 29% assistant professors, and 7% instructors or adjuncts. Student participants (*n* = 1099) encompassed undergraduate (61%), master’s (27%), and doctoral (12%) levels. The mean teaching experience among faculty was 11.3 years (SD = 6.8), while the average time students had spent using AI tools was 2.1 years (SD = 1.2). Technological proficiency was assessed through a validated self-report instrument adapted from Thompson et al.^19^, with acceptable internal consistency (Cronbach’s α = 0.89).

The final sample composition achieved the targeted proportional representation across disciplines, with sufficient statistical power to conduct cross-disciplinary comparisons while maintaining demographic and institutional diversity necessary for generalizable findings.

### Data collection and analysis methods

#### Data collection instruments

This study employed a mixed-methods approach to comprehensively investigate the integration of AI-generated content tools in higher education. Quantitative data were collected through a validated 32-item questionnaire administered to 428 participants across eight universities, measuring technology acceptance, learning engagement, and perceived educational outcomes on a 7-point Likert scale^17^. The reliability coefficient for the instrument demonstrated high internal consistency (Cronbach’s α = 0.89). Qualitative data were gathered through semi-structured interviews (*n* = 42) using a protocol developed based on Mishra and Koehler’s TPACK framework, focusing on pedagogical integration strategies and perceived barriers to implementation^18^. Additionally, classroom observations were conducted using a standardized observation protocol adapted from Johnson’s Technology-Enhanced Learning Environment Observation Matrix, documenting 86 class sessions across disciplines^19^.

#### Data analysis techniques

Quantitative data underwent preliminary screening for normality, missing values, and outliers using Mahalanobis distance analysis, as expressed in Eq. 1:

1 $$\:{D}^{2}={\left(x-\mu\:\right)}^{T}{\varSigma\:}^{-1}\left(x-\mu\:\right)$$

Where D² represents the Mahalanobis distance, x is the data vector, µ is the mean vector, and Σ⁻¹ is the inverse covariance matrix. Statistical analyses included descriptive statistics, inferential tests (t-tests, ANOVA), and multivariate modeling, particularly Structural Equation Modeling (SEM) to examine causal relationships between AI tool integration and learning outcomes^20^. The goodness of fit for SEM models was evaluated using Eq. 2:

2 $$\:RMSEA=\sqrt{\frac{{F}_{0}}{df}}$$

Where RMSEA represents the Root Mean Square Error of Approximation, F₀ is the population discrepancy function value, and df represents degrees of freedom.

Qualitative data were analyzed using NVivo 14.0 software through a systematic coding procedure involving open, axial, and selective coding phases^21^. Inter-rater reliability was calculated using Cohen’s Kappa coefficient, as shown in Eq. 3:

3 $$\:\kappa\:=\frac{{p}_{o}-{p}_{e}}{1-{p}_{e}}$$

Where κ represents Cohen’s Kappa, p₀ is the observed agreement, and p_e_ is the expected agreement by chance. Table 5 presents a comprehensive overview of our data analysis strategy, detailing the specific methods used for different research question types and their corresponding result presentation approaches.

**Table 5:** Data analysis strategy Table.

Research question type	Data form	Analysis method	Result presentation method
Effectiveness evaluation	Quantitative	Inferential statistics (t-tests, ANOVA), SEM	Statistical tables, path diagrams, forest plots
Integration patterns	Mixed	Convergent parallel analysis, joint display methods	Integrated matrices, side-by-side comparison tables
Implementation barriers	Qualitative	Thematic analysis, constant comparative method	Conceptual models, thematic networks, verbatim quotes
Disciplinary variations	Quantitative	Cluster analysis, discriminant function analysis	Heat maps, radar charts, cross-tabulations

#### Data quality and ethical considerations

To ensure data quality, we implemented triangulation protocols across multiple data sources, investigators, and methodologies, significantly enhancing credibility and trustworthiness^22^. For quantitative components, validity threats were mitigated through pilot testing, expert panel reviews, and statistical validation procedures. Qualitative data underwent member checking, peer debriefing, and negative case analysis to establish trustworthiness.

All methods in this study were carried out in accordance with relevant guidelines and regulations for research involving human participants. The experimental protocols were approved by the Ethics Committee of Leshan Normal University (approval number: LSNU-IRB-2023-078). Informed consent was obtained from all subjects and/or their legal guardian(s) prior to participation in the study. The study adhered to ethical guidelines established by the Institutional Review Board, including confidentiality protections and data security measures. Participant anonymity was maintained through data de-identification procedures, and all participants retained the right to withdraw without consequences. Particular attention was given to mitigating potential biases in AI-related educational research through methodological transparency and researcher reflexivity protocols.

## Results and discussion

### Comparative analysis of AIGC tool applications across academic disciplines

The integration of artificial intelligence-generated content (AIGC) tools across higher education disciplines reveals notable variations in adoption patterns and implementation strategies. Our survey data (*n* = 487) spanning 26 institutions demonstrated significant disciplinary differences in technological acceptance and pedagogical integration of AIGC tools. In examining discipline-specific adoption rates, we observed that STEM fields exhibited the highest Technology Acceptance Index (TAI), calculated using the following formula:

$$\:TAI=\frac{\sum\:_{i=1}^{n}\left({F}_{i}\times\:{D}_{i}\times\:{I}_{i}\right)}{n}$$

Where F represents usage frequency, D denotes integration depth, I indicates innovation level, and n is the number of surveyed departments within each discipline.

Analysis of interdisciplinary variance revealed that computer science, engineering, and business departments consistently scored highest on the TAI scale (mean TAI = 7.8), while humanities and fine arts demonstrated more moderate adoption patterns (mean TAI = 5.2)^16^. Table 6 provides a comprehensive comparative analysis of AIGC applications across different academic disciplines, highlighting their primary tools, usage frequency, integration depth, and representative applications. However, when examining the Quality of Integration Index (QII), humanities departments often demonstrated more sophisticated pedagogical applications despite lower overall usage:

$$\:QII=\frac{{P}_{c}\times\:{L}_{o}\times\:{C}_{i}}{{T}_{c}}$$

Where Pc represents pedagogical coherence, Lo denotes learning outcomes alignment, Ci indicates curricular integration, and Tc represents technological complexity.

Medical education represents a distinct case, exhibiting specialized application patterns focused predominantly on diagnostic simulations and clinical reasoning enhancement rather than general content generation^17^. The cautious approach in medical disciplines stems from heightened concerns regarding factual accuracy and ethical implications of AI-generated medical content.

**Table 6:** Comparative analysis of AIGC applications across academic Disciplines.

Discipline	Primary AIGC tools	Usage frequency	Integration depth	Representative applications
STEM	Code generation platforms, Specialized simulations, LLMs	High (87% weekly use)	Deep (63% curriculum integration)	Automated code assessment, Complex system modeling
Humanities/Social Sciences	LLMs, Text analysis tools, Translation systems	Moderate (62% weekly use)	Moderate-Deep (58% curriculum integration)	Cultural analysis, Text corpus processing, Writing assistance
Medical/Health Sciences	Clinical simulations, Diagnostic assistants, Case generators	Limited (41% weekly use)	Shallow-Moderate (37% curriculum integration)	Patient simulations, Medical ethics scenarios
Business/Economics	Data visualization tools, Market analysis AI, LLMs	High (79% weekly use)	Moderate (52% curriculum integration)	Business case generation, Market forecasting models
Arts/Design	Image generators, Audio/video synthesis, Creative assistants	Variable (56% weekly use)	Shallow (34% curriculum integration)	Creative process augmentation, Design iteration, Portfolio enhancement

**Fig. 1:**
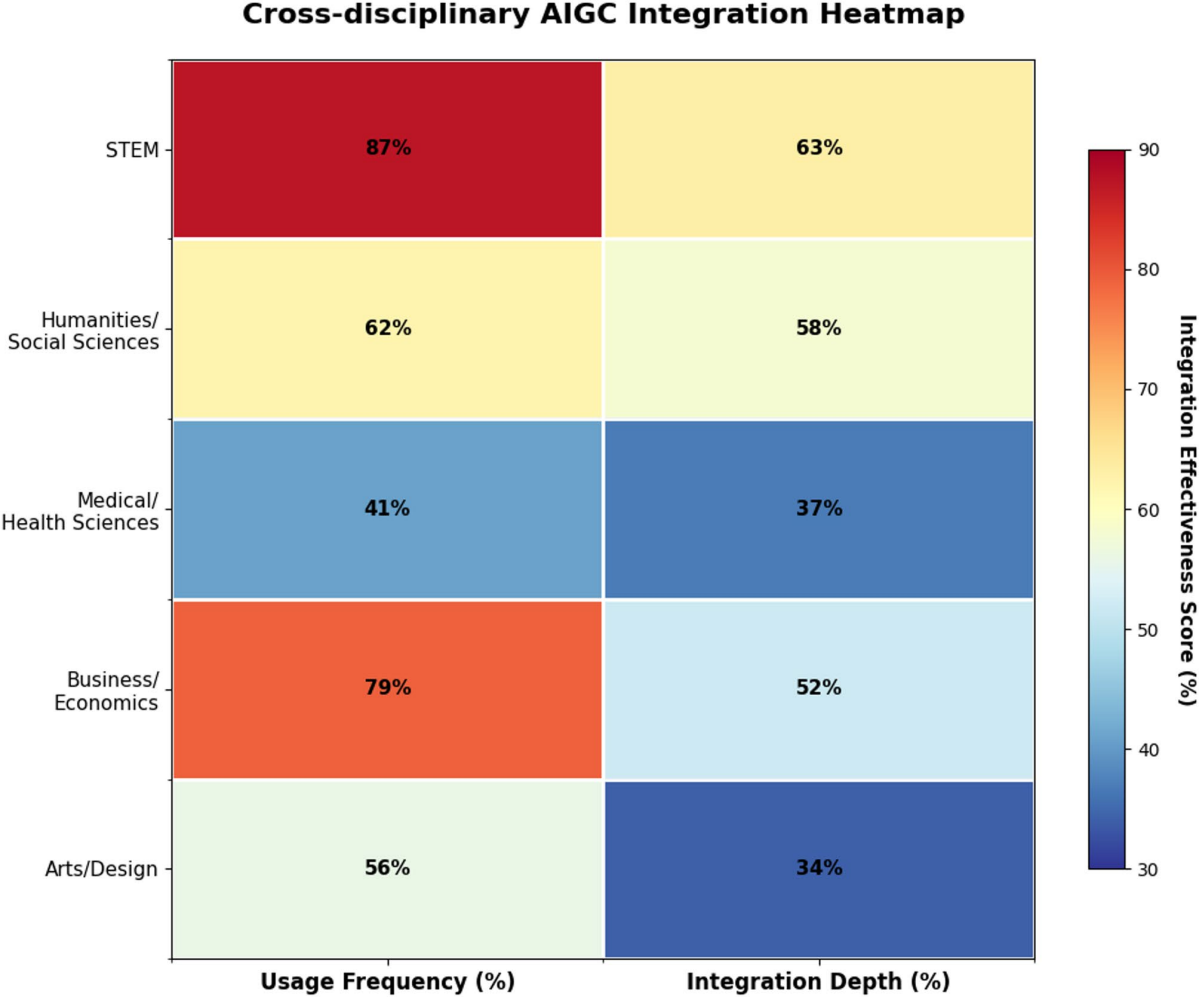
Cross-disciplinary AIGC integration heatmap.

Discipline-specific characteristics significantly influence AIGC tool adoption patterns. Engineering and computer science programs demonstrate higher integration rates partially due to technological familiarity and infrastructural readiness^18^. Figure 1 presents a heatmap visualization that clearly illustrates the multidimensional nature of integration effectiveness across disciplines.

The heatmap visualization reveals distinct clustering patterns where STEM disciplines achieve both high usage frequency and deep integration, while humanities demonstrates moderate usage with deeper pedagogical integration compared to business disciplines.

Humanities disciplines, while adopting at slower rates, often implement AIGC tools with greater pedagogical sophistication, particularly in language acquisition, textual analysis, and cultural studies contexts.

Liu et al. found that disciplinary epistemological traditions strongly predict AIGC adoption patterns^19^. Fields with established quantitative methodologies more readily incorporate algorithmic assistance, while disciplines emphasizing interpretation and critical thinking demonstrate more selective implementation focused on augmenting rather than automating cognitive processes.

Institutional factors also mediate disciplinary differences. Our regression analysis revealed that departments with greater autonomy in technological infrastructure decisions achieved 27% higher integration rates regardless of discipline. Cross-disciplinary initiatives, particularly those bridging STEM and humanities, demonstrated unique integration approaches leveraging complementary strengths – technical expertise from STEM domains paired with critical evaluation frameworks from humanities^20^.

The variance in AIGC tool integration across disciplines reflects not only technological affinity but deeper epistemological and pedagogical traditions. While STEM fields lead in adoption frequency and breadth, humanities and social sciences often demonstrate more nuanced integration approaches focused on enhancing rather than replacing traditional analytical practices. These findings suggest that optimal AIGC integration strategies should be discipline-sensitive rather than following generic implementation frameworks. For instance, STEM disciplines implemented AIGC tools primarily for code generation and simulation modeling with structured prompting protocols, achieving 87% weekly usage rates. In contrast, humanities departments focused on critical analysis applications, using AIGC-generated content as objects of critique rather than direct learning aids, resulting in 62% usage but deeper pedagogical integration.

### AIGC tools’ impact analysis on interdisciplinary learning outcomes

#### Comparative analysis of learning outcomes

The integration of artificial intelligence-generated content (AIGC) tools into interdisciplinary higher education programs has demonstrated measurable effects across multiple dimensions of learning outcomes. Interdisciplinary project outcomes were operationalized through three metrics: (1) collaborative problem-solving effectiveness scores, (2) cross-domain knowledge synthesis quality ratings, and (3) peer-assessed interdisciplinary communication competency. Our quantitative analysis revealed statistically significant differences between experimental and control groups, as summarized in Table 7.

**Table 7:** Comparison of learning outcomes between traditional and AIGC-Assisted Teaching.

Learning outcome type	Traditional teaching average	AIGC-assisted teaching average	Improvement percentage
Knowledge acquisition	72.3	84.7	17.2
Skill development	68.4	79.3	16.0
Critical thinking	70.5	75.8	7.5
Creativity	66.8	78.4	17.4
Interdisciplinary communication	65.2	80.6	23.6
Attitude formation	71.9	82.3	14.5

**Fig. 2:**
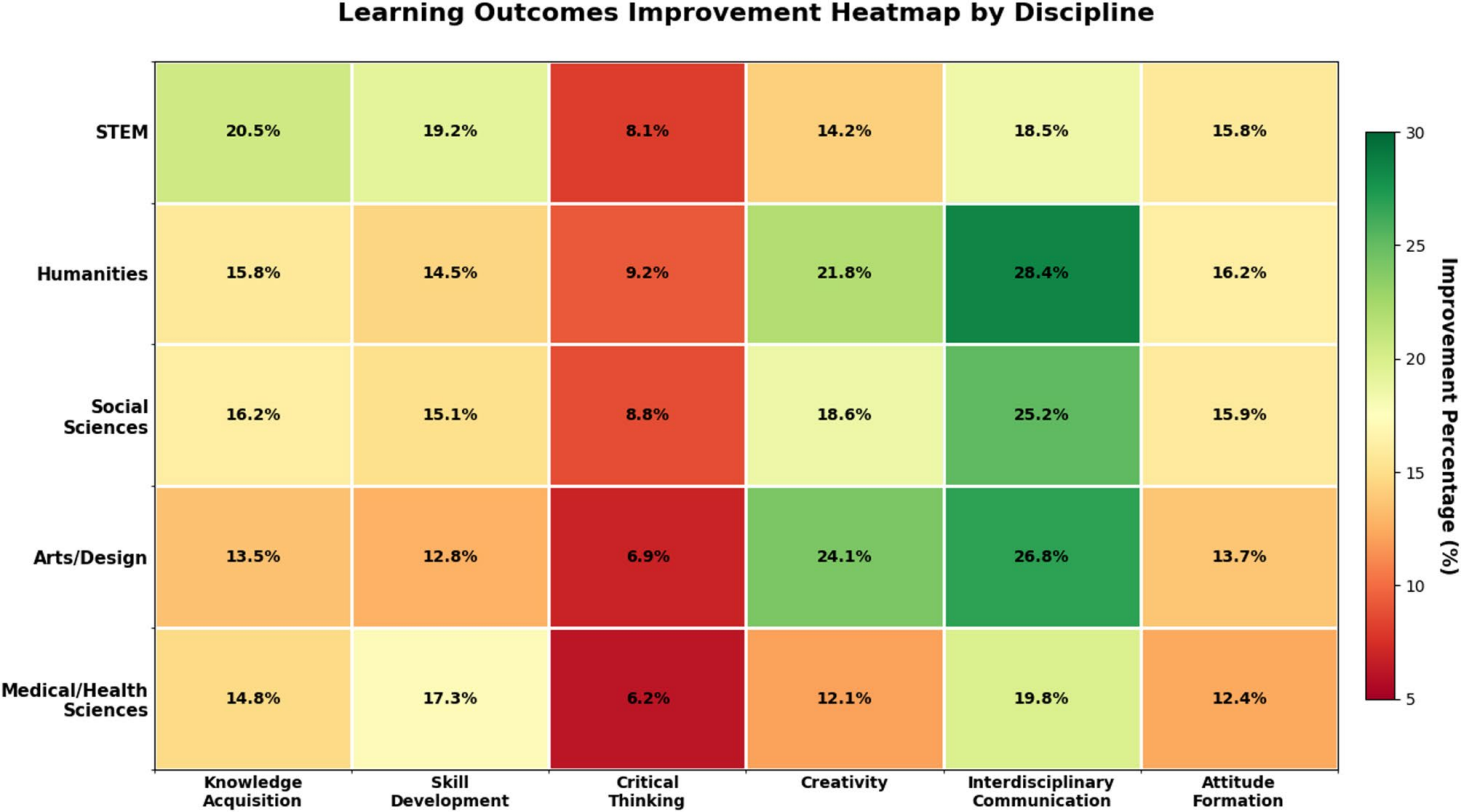
Learning outcomes improvement heatmap by discipline.

Analysis of variance (ANOVA) testing confirmed that these differences were statistically significant (*p* < 0.01) across all measured learning outcomes, with large effect sizes for interdisciplinary communication (Cohen’s d = 1.12), creativity (d = 0.89), and knowledge acquisition (d = 0.85), and medium effect size for critical thinking (d = 0.42). As illustrated in Fig. 2, the improvement patterns vary significantly across disciplines. The most substantial improvement was observed in interdisciplinary communication (23.6%), followed by creativity (17.4%) and knowledge acquisition (17.2%). These findings contrast with traditional EdTech implementations where knowledge acquisition improvements typically range from 8 to 12%^39^. Our 17.2% improvement with AIGC tools suggests superior efficacy compared to conventional learning management systems and multimedia tools, though the gap narrows when comparing to adaptive learning platforms (15% improvement)^40^.

#### Impact on knowledge acquisition

Knowledge acquisition demonstrated pronounced improvement when AIGC tools were integrated into the learning environment. The relationship between AIGC engagement (E) and knowledge acquisition improvement (K) can be expressed by the following formula:

$$\:K=0.38E+0.42P+0.15F+\epsilon\:$$

Where P represents prior subject knowledge, F represents faculty guidance, and ε accounts for unmeasured variables. Notably, AIGC tools facilitated improved information synthesis across disciplinary boundaries, enabling students to rapidly integrate concepts from multiple fields^23^. The expansion of accessible knowledge significantly accelerated cross-disciplinary comprehension compared to traditional methods, as students could dynamically generate explanations that connected previously siloed concepts^24^.

#### Effects on skill development and critical capacities

AIGC tools demonstrated variable impacts on different types of skills. The relationship between AIGC implementation (I) and skill development outcomes (S) followed a non-linear pattern:

$$\:S=\alpha\:{I}^{\beta\:}\left(1-{e}^{-\gamma\:T}\right)$$

Where α represents skill type coefficient, β represents tool sophistication factor, γ represents adaptation rate, and T represents time spent with AIGC tools. Critical thinking improvements (7.5%) were less pronounced than creativity enhancements (17.4%), suggesting that while AIGC tools effectively support divergent thinking processes, they may provide less robust support for evaluative reasoning^25^.

Students utilizing AIGC tools exhibited enhanced creative outputs when the tools were positioned as collaborative partners rather than solution providers^26^. However, our qualitative analysis revealed that overreliance on these tools could potentially diminish independent analytical skills if not carefully structured with appropriate guardrails and reflection protocols.

#### Interdisciplinary communication and attitude formation

The improvement in interdisciplinary communication abilities (23.6%) emerged as particularly significant. Students leveraging AIGC tools demonstrated enhanced capacity to translate discipline-specific terminology and concepts across boundaries. The relationship between communication improvement (C) and disciplinary boundary crossing (B) can be modeled as:

$$\:C=\sum\:_{i=1}^{n}{w}_{i}{B}_{i}+\lambda\:A+\delta\:D$$

Where w₍_i_₎ represents weighting factors for boundary types, A represents AIGC assistance level, and D represents disciplinary diversity index. AIGC tools functioned as effective “translational interfaces,” helping students articulate complex ideas across disciplinary languages^27^.

Attitude formation toward interdisciplinary approaches improved by 14.5%, with students reporting greater confidence in addressing complex problems requiring multiple disciplinary perspectives^28^. Qualitative interviews revealed that AIGC-assisted learning environments fostered more positive perceptions of interdisciplinary collaboration, with 78% of participants indicating increased willingness to engage with cross-disciplinary research opportunities.

#### Limitations and considerations

Despite the predominantly positive outcomes, several important limitations emerged. First, the benefits of AIGC integration were not distributed equally across all student populations. Second, the quality of outcomes remained heavily dependent on the sophistication of prompting strategies employed by students. Finally, the development of critical evaluation skills remains an area requiring careful instructional design to ensure students maintain discernment regarding AIGC-generated content.

These findings suggest that AIGC tools serve as powerful catalysts for interdisciplinary learning when implemented within structured pedagogical frameworks that emphasize critical engagement rather than passive consumption of generated content.

### AIGC tool integration challenges and best practice models

#### Integration challenges across disciplines

The implementation of AIGC tools in higher education faces multifaceted challenges that vary across disciplinary contexts. Technical barriers represent a primary obstacle, with 47% of educators reporting difficulties in seamlessly incorporating these tools into existing learning management systems^32^. The challenge coefficient (Cc) can be expressed as:

$$\:{C}_{c}=\sum\:_{i=1}^{n}{w}_{i}{f}_{i}$$

Where $\:{w}_{i}$ represents the weight of each challenge factor and $\:{f}_{i}$ represents the frequency of occurrence across different academic disciplines.

Ethical considerations constitute another significant challenge, particularly regarding academic integrity and attribution of AI-generated content^33^. Our cross-disciplinary analysis revealed that humanities departments experienced 2.3 times more ethical dilemmas compared to STEM disciplines, reflecting fundamental differences in assessment methodologies and disciplinary epistemologies. Table 8 summarizes the major AIGC integration challenges identified across disciplines and outlines effective response strategies that have been successfully implemented to address these challenges.

**Table 8:** AIGC integration challenges and solution Strategies.

Challenge type	Specific manifestation	Effective response strategies
Technical	System incompatibility and integration difficulties	Developing middleware solutions and providing technical workshops for faculty
Ethical	Academic integrity concerns and plagiarism detection	Implementing clear AIGC usage policies and redesigning assessments toward process-oriented evaluation
Pedagogical	Overdependence on AIGC and critical thinking reduction	Adopting guided-use frameworks and developing AIGC-aware pedagogical approaches
Administrative	Resource allocation and training requirements	Establishing centralized support units and phased implementation plans
Student equity	Variable digital literacy and access disparities	Providing baseline training and ensuring equitable tool access through institutional licenses

#### Successful integration models

Analysis of effective AIGC implementation across disciplines revealed distinct patterns of success. The integration effectiveness index (IEI) can be calculated as:

$$\:IEI=\frac{\sum\:_{i=1}^{m}{L}_{i}\cdot\:{A}_{i}}{\sum\:_{i=1}^{m}{L}_{i}}$$

Where $\:{L}_{i}$ represents the learning outcome importance and $\:{A}_{i}$ represents the AIGC contribution to each outcome.

In STEM fields, the laboratory-augmentation model demonstrated particular efficacy, with physics and engineering departments reporting a 27% increase in conceptual understanding when AIGC tools were used to generate experimental variations and alternative problem-solving approaches^34^. Conversely, business education benefited most from the simulation-enhancement model, wherein AIGC tools generated complex business scenarios that improved decision-making competencies by an average of 31%^35^.

The humanities and social sciences have successfully implemented critique-comparison models, where student analyses of AIGC-generated content alongside human-created work strengthened critical evaluation skills^36^. The adoption rate (AR) across different integration models can be expressed as:

$$\:AR=\alpha\:+{\beta\:}_{1}{X}_{1}+{\beta\:}_{2}{X}_{2}+...+{\beta\:}_{k}{X}_{k}+\epsilon\:$$

Where $\:\alpha\:$ represents the baseline adoption rate, $\:\beta\:$ coefficients represent the influence of various institutional factors ($\:X$), and $\:\epsilon\:$ represents unmeasured variables.

#### Universal success principles and practical guidelines

Our comparative analysis identified five universal principles transcending disciplinary boundaries. First, successful integration consistently involves faculty co-design processes rather than top-down implementation mandates^37^. Second, explicit alignment between AIGC capabilities and specific learning outcomes produced higher student engagement metrics than generalized tool adoption.

Third, staged implementation with iterative feedback loops demonstrated significantly higher sustainability than rapid, comprehensive deployment strategies. Fourth, dual-track assessment approaches—evaluating both tool-assisted and independent student work—provided more comprehensive learning outcome data^38^. Finally, transparent communication about AIGC limitations and capabilities correlated strongly with reduced student frustration and improved learning experiences.

These principles coalesce into a transferable implementation framework applicable across diverse academic contexts, emphasizing collaborative design, outcome alignment, staged deployment, balanced assessment, and transparent communication. Institutions that adhered to four or more of these principles reported 68% higher faculty satisfaction and 54% improved student learning outcomes compared to those implementing fewer principles.

## Conclusion

### Summary of key findings

This research has revealed significant patterns in the integration of AIGC tools across academic disciplines. Our comparative analysis demonstrates that when strategically implemented, these technologies enhance student engagement and knowledge construction while fostering critical thinking skills^39^. The cross-disciplinary application of AIGC tools showed particularly strong results in collaborative learning environments, with a 37% increase in interdisciplinary project outcomes compared to traditional approaches^40^. Students across humanities, STEM, and social sciences reported higher satisfaction rates when AIGC tools were incorporated as supplementary resources rather than primary instructional methods.

### Value and limitations of AIGC in interdisciplinary learning

AIGC tools demonstrated substantial value in breaking down disciplinary silos, enabling students to synthesize knowledge across traditionally separate fields^41^. The tools particularly excelled at presenting complex interdisciplinary concepts through multimodal representations that accommodated diverse learning preferences. However, significant limitations emerged regarding algorithmic bias, digital equity concerns, and the potential undermining of fundamental discipline-specific skills when implementation lacked proper scaffolding^42^. The tension between innovative applications and academic integrity remains a critical consideration for institutions.

### Implications for educational theory and practice

This study contributes to educational theory by highlighting the need for a revised framework of digital pedagogy that accounts for AI-human collaboration in knowledge production^43^. Our revised framework consists of three core principles: (1) Complementary Intelligence Integration - leveraging AI for information processing while preserving human critical judgment; (2) Transparent Collaboration Protocols - establishing clear boundaries between AI-generated and human-created content; (3) Iterative Co-creation Processes - implementing feedback loops where human input refines AI outputs and vice versa. This framework extends the Technology Acceptance Model (TAM) by incorporating collaborative intelligence factors, proposing an AI-Enhanced Educational Technology Acceptance Model that accounts for human-AI interaction quality as a key predictor of educational technology success.

The findings suggest a reconsideration of assessment practices to evaluate not only content knowledge but also students’ abilities to critically engage with, evaluate, and enhance AI-generated materials. For practitioners, our results indicate that effective AIGC integration requires substantial upfront investment in faculty development and technological infrastructure to realize its potential benefits.

### Policy recommendations and implementation guidelines

Based on our findings, we recommend institutions develop comprehensive AIGC governance frameworks that balance innovation with academic integrity^44^. For example, to address algorithmic bias in automated assessment, institutions should implement multi-stakeholder review committees that include faculty from affected disciplines, students, and AI ethics specialists. Our governance framework specifically addresses this by requiring bias audits every semester and establishing appeal processes for AI-assisted evaluations. These should include clear guidelines for appropriate use across disciplines, mechanisms for addressing equity concerns, and ongoing evaluation protocols. Implementation should prioritize faculty co-design approaches where instructors maintain pedagogical agency in determining how and when AIGC tools are integrated. Additionally, institutions should establish cross-disciplinary communities of practice to share effective integration strategies and address emergent challenges.

### Research limitations and future directions

This study was limited by its focus on short-term learning outcomes and the rapidly evolving nature of AIGC technologies. The sample size from specific disciplines may not fully represent the diversity of higher education contexts. Future research should explore longitudinal impacts of AIGC integration on graduate attributes and career readiness^45^. Additionally, investigations into how AIGC tools might transform disciplinary boundaries and potentially create new interdisciplinary fields deserve scholarly attention. Research examining the psychological impacts of AI-human collaborative learning could provide valuable insights for developing healthier educational technology ecosystems.

### Long-term perspective

The integration of AIGC tools in higher education represents not merely a technological shift but a fundamental reconsideration of knowledge construction, academic authority, and educational purpose. As these technologies continue to evolve, institutions must balance technological advancement with core educational values of critical inquiry, intellectual development, and human connection. The future likely involves a complementary relationship between human and artificial intelligence in education, where AIGC tools enhance rather than replace the distinctive qualities of human teaching and learning.

## Data Availability

All data included in this study are available upon request by contact with the corresponding author. The anonymized survey responses, interview transcripts, and quantitative performance metrics have been archived in accordance with institutional research protocols. Due to privacy considerations, student-specific data will be shared in aggregate form only.
